# Quantitative lipidomic analysis of mouse lung during postnatal development by electrospray ionization tandem mass spectrometry

**DOI:** 10.1371/journal.pone.0203464

**Published:** 2018-09-07

**Authors:** Srikanth Karnati, Vannuruswamy Garikapati, Gerhard Liebisch, Paul P. Van Veldhoven, Bernhard Spengler, Gerd Schmitz, Eveline Baumgart-Vogt

**Affiliations:** 1 Institute for Anatomy and Cell Biology II, Division of Medical Cell Biology, Justus Liebig University Giessen, Giessen, Germany; 2 Institute of Inorganic and Analytical Chemistry, Justus Liebig University Giessen, Giessen, Germany; 3 Institute for Clinical Chemistry and Laboratory Medicine, University Hospital Regensburg, Regensburg, Germany; 4 Laboratory for Lipid Biochemistry and Protein Interactions, Campus Gasthuisberg, KU Leuven, Leuven, Belgium; Center of Pediatrics, GERMANY

## Abstract

Lipids play very important roles in lung biology, mainly reducing the alveolar surface tension at the air-liquid interface thereby preventing end-expiratory collapse of the alveoli. In the present study we performed an extensive quantitative lipidomic analysis of mouse lung to provide the i) total lipid quantity, ii) distribution pattern of the major lipid classes, iii) composition of individual lipid species and iv) glycerophospholipid distribution pattern according to carbon chain length (total number of carbon atoms) and degree of unsaturation (total number of double bonds). We analysed and quantified 160 glycerophospholipid species, 24 sphingolipid species, 18 cholesteryl esters and cholesterol from lungs of a) newborn (P1), b) 15-day-old (P15) and c) 12-week-old adult mice (P84) to understand the changes occurring during postnatal pulmonary development. Our results revealed an increase in total lipid quantity, correlation of lipid class distribution in lung tissue and significant changes in the individual lipid species composition during postnatal lung development. Interestingly, we observed significant stage-specific alterations during this process. Especially, P1 lungs showed high content of monounsaturated lipid species; P15 lungs exhibited myristic and palmitic acid containing lipid species, whereas adult lungs were enriched with polyunsaturated lipid species. Taken together, our study provides an extensive quantitative lipidome of the postnatal mouse lung development, which may serve as a reference for a better understanding of lipid alterations and their functions in lung development and respiratory diseases associated with lipids.

## Introduction

The lung is composed of more than 40 different pulmonary cell types, whose cellular membranes are enriched with lipids that perform a variety of functions including maintenance of the lung architecture [1–3]. In addition, alveolar epithelial type II cells of the pulmonary epithelium that are lining the alveolar surface synthesize and secrete surfactant into the alveolar space. Pulmonary surfactant is a complex mixture of lipids (phospholipids, triglycerides, fatty acids and cholesterol, etc.), surfactant proteins (A-D) and a small amount of carbohydrates. The majority of the pulmonary lipids comprise glycerophospholipids (GP) in which phosphatidylcholine (PC) is a predominant lipid class making up to 50% of the phospholipids. Phosphatidylethanolamine (PE) makes up to 20% of the lipids and phosphatidylserine (PS), phosphatidylinositol (PI) and phosphatidylglycerol (PG) constitute 12%-15% of the total phospholipid pool [4, 5].

Pulmonary lipids are important and diverse biomolecules that are involved in many biological processes. The thus far known functions of the major lung lipids include 1) prevention of alveolar collapse and preservation of bronchiolar patency [6], 2) improvement of mucociliary transport [7], 3) involvement in innate immunity and viral protection [8, 9], 4) action as potent intracellular signalling molecules in lung inflammation [10], 5) involvement of lipid mediators like leukotrienes, lipoxins and prostaglandins in specific reactions of inflammation and immunity [11, 12], 6) suppression of the proliferation, immunoglobulin production and cytotoxicity of lymphocytes [6]. In fact, alterations in whole lung lipid composition and/or deficiency of pulmonary surfactant lipids are closely associated with a) respiratory distress syndrome (RDS) [13], b) bronchopulmonary dysplasia (BPD) [13, 14], c) asthma [15], d) chronic obstructive pulmonary disease (COPD) [15, 16], e) cystic fibrosis [17], f) pneumonia [17, 18], g) lung injury [19], h) cancer [20] and in other lung diseases [6, 21].

High heterogeneity of lung tissue (e.g., bronchial versus alveolar regions) and differences in the lipid composition of individual pulmonary cell types create a complex mixture of lipid classes and molecular species associated with a set of potential complications. Despite these difficulties, several studies analyzed the lipid composition of lung tissue in different mammalian species such as in pig [22], rat [23], rabbit [24], monkey [24], dog [25], bovine [26], mice [27] and human [28] and also compared the lung lipid composition among different mammalian species [28, 29]. Actually, the major lipid classes were similar among different mammalian species and minor differences were observed only in the class of phospholipids. Interestingly, surfactant has proven to be highly diverse across species in its molecular design, especially in the concentration of individual surfactant proteins and its GP profile [30, 31]. Further, most of these studies focused on the analysis of PC, which are the prominent class of lipids in whole lung tissue and pulmonary surfactant. Furthermore, among the analyzed PC, dipalmitoylphosphatidylcholine (DPPC; PC 32:0), a species with two saturated acyl chains, is believed to be a major compound of the pulmonary surfactant [32]. However, recent studies on homeothermic/heterothermic mammalian species surfactant showed that DPPC is not the only major surfactant phospholipid component. In addition to major PC lipid molecular species, there are minor lipid classes such as PG and PI, which also play an important role in lung biology [33, 34].

Lung development in the majority of mammalian species (e.g., rat, mice and humans) continues postnatally. One important aspect of postnatal lung development is alveolarization, a process, in which the total number of terminal gas exchange units increase total size and surface area of the lung [35–37]. To address the lipidomic changes in the fetal and postnatal lung development, various studies were conducted in several mammalian species (e.g., rat, rabbit, lamb, pig and human) and in birds (e.g., duck and chicken) by using morphometric and biochemical approaches [35–41]. However, the existing lipidomic studies of postnatal lung development primarily focused on the composition of surfactant PC and very few PG lipid species [31, 40, 41]. Dautel et al., recently reported postnatal developmental changes of mouse lung using a multi-omics approach [27]. These data are consistent with our results, but in comparison to their study (day7, day14 and 6–8 week old animals), we have used a wider time frame between birth (new born) and the 12^th^ week of life. Furthermore, the highly abundant cholesterol, as well as cholesteryl ester species were not analysed in the Dautel et al., study during postnatal development. Moreover, we performed direct infusion lipidomics using triple-quadrupole MS analytical setup and provided the quantitative information (nmol/mg wet weight) of individual lipid species of possible major lipid classes during postnatal development.

In our study, we employed electrospray ionization tandem mass spectrometry (ESI-MS/MS) to 1) investigate total lipid quantity and 2) to perform a detailed analysis of lipid classes (PC, LPC, PG, PS, PE, PE P, PI, SM, Cer, HexCer, CE, and cholesterol), and 3) the composition of individual lipid species (e.g. PC 32:0, PC 32:1 etc.), and 4) to analyse their distribution pattern based on the carbon chain length (total number of carbon atoms). Additionally, we analysed the degree of unsaturation (total number of double bonds) of whole lung homogenates in newborn, 15-day-old and 12-week-old adult mice in order to provide a detailed lipidomic information during the postnatal development. Our current study provides an extensive quantitative lipidome of mouse whole lung, which may serve as a reference for a better understanding of the development of lung and molecular mechanisms underlying various pulmonary diseases associated with the lipid alterations.

## Materials and methods

### Materials

Unless otherwise mentioned, all chemicals were procured from Sigma-Aldrich (Deisenhofen, Germany). Phospholipid standards were obtained from Avanti Polar Lipids (Alabaster, AL, USA). Cholesterol and cholesteryl ester standards of purity greater than 95% were obtained from Sigma (Taufkirchen, Germany). High purity cholesterol-(25, 26, 26, 26, 27, 27, 27-d7) was purchased from Cambridge Isotope Laboratories (Andover, MA, USA). HPLC grade solvents methanol and chloroform were obtained from Merck (Darmstadt, Germany). Analytical grade ammonium acetate and acetyl chloride were obtained from Sigma-Aldrich (Buchs, Switzerland). All other reagents used were of high purity and analytical grade.

### Animal experiments

Twelve-week-old adult male mice, 15-day-old males and pregnant females of C57BL/6J genetic background were obtained from Charles River, Sulzfeld, Germany. Mice were kept on a normal laboratory diet and water *ad libitum* and housed in cages under standardized environmental conditions (12 hours light/dark cycle, 23°C ± 1°C and 55% ± 1% relative humidity) at the central animal facility of the Justus Liebig University Giessen, Germany. After delivery of the newborn pups in the morning, they were taken directly out of the animal facility. 15-day-old and 12-week-old adult mice were killed by cervical dislocation and the newborn pups were killed by decapitation. All experiments with laboratory mice were approved by the governmental ethics committee for animal welfare (Regierungspräsidium Giessen, Germany, permit number: V 54–19 C 20/15 c GI 20/23).

### Lipid extraction and sample preparation

The newborn (P1), 15-day-old (P15) and 12-week-old adult (P84) male mice fur was vertically incised from the pelvis to the mandibles and removed to both sides. The abdomen was opened and a bilateral pneumothorax was produced by puncturing the abdominal surface of the diaphragm. The sternum was cut in the middle and the thorax was opened with a thorax spanner. The lungs were isolated carefully and snap-frozen immediately.

Fresh snap-frozen lungs from P1, P15 and P84 male mice were homogenized with a Precellys homogenator (peQlab Biotech GmbH, Erlangen, Germany) at a concentration of 50 μg wet weight per μL. Homogenate corresponding to 2 mg wet weight was used for extraction, and lipids were extracted according to the procedure described by Bligh and Dyer [42]. Upon phase separation, the chloroform phase was transferred to a fresh tube and dried under a stream of nitrogen gas. For each lipid class (except for SM and PE P), two naturally not occurring lipid species were added as internal standards, to compensate for variations in sample preparation and ionization efficiency. PC 14:0/14:0 (28:0), PC 22:0/22:0 (44:0) for PC and SM, PE 14:0/14:0 (28:0), PE 20:0/20:0 (40:0) for PE and PE based plasmalogens (PE P), PS 14:0/14:0 (28:0), PS 20:0/20:0 (40:0), PG 14:0/14:0 (28:0), PG 20:0/20:0 (40:0), PI 16:0/16:0 (32:0), LPC 13:0, LPC 19:0, Cer 14:0, Cer 17:0 (d18:1/17:0), cholesterol-d7, CE 17:0 and CE 22:0 internal standards were added for the analysed lipid classes.

### Mass spectrometric analysis of lipids

Lung homogenates were subjected to lipidome analysis by electrospray ionization-tandem mass spectrometry (ESI-MS/MS) in positive-ion mode as described [43–46]. Briefly, the samples were analyzed on a triple-quadrupole mass spectrometer (Quattro Ultima, Micromass, Manchester, UK) by direct flow injection analysis using an autosampler (HTS PAL, Zwingen, Switzerland) and a binary pump (Model 1100, Agilent, Waldbronn, Germany) with a solvent mixture of methanol containing 10 mM ammonium acetate and chloroform (3:1, v/v). A flow gradient was performed starting with a flow of 55 μL/min for 6 seconds followed by 30 μL/min for 1 minute and an increase to 250 μL/min for another 12 seconds. The mass spectrometer was equipped with electrospray ionization and operated in positive-ion mode using following tune parameters as capillary voltage, 3.5 kV; cone voltage, 110 V; collision energy, 30 V; collision gas, argon at a pressure of 0.13 Pa.

A precursor ion of *m/z* 184, which is specific for phosphocholine-containing lipids, was used for the analysis of phosphatidylcholine (PC), lysophosphatidylcholine (LPC) and sphingomyelin (SM) lipid species [43, 44]. Neutral loss scans of 141 and 185 were used for the phosphatidylethanolamine (PE) and phosphatidylserine (PS) respectively [47]. Fragment ions of *m/z* 364, 390, and 392 were used for the quantification of PE P-16:0, PE P-18:1 and PE P-18:0 plasmalogens according to Zemski et al. [48]. Neutral loss scans of 189 and 277 were used for the ammonium adducts of phosphatidylglycerol (PG) and phosphatidylinositol (PI) respectively [49]. Sphingosine (d18:1) based ceramides (Cer) were analysed using a fragment ion of *m/z* 264 [45]. Cholesterol and cholesteryl esters (CE) were quantified using a fragment ion of *m/z* 369 after selective derivatization of cholesterol using acetyl chloride [46].

After identification of relevant lipid species, selective ion monitoring analysis was performed to increase precision of the analysis of lipids. Quantification of different classes of lipid species was achieved by plotting the standard calibration curves of naturally occurring lipid species of PC 34:1, 36:2, 38:4, 40:0 and PC O-16:0/20:4; SM d18:1/16:0, 18:1, 18:0; LPC 16:0, 18:1, 18:0; PE 34:1, 36:2, 38:4, 40:6 and PE P-16:0/20:4; PS 34:1, 36:2, 38:4, 40:6; Cer d18:1/16:0, 18:0, 20:0, 24:1, 24:0; cholesterol, CE 16:0, 18:2, 18:1, 18:0. Correction of isotopic overlap of lipid species as well as data analysis were performed using self-programmed Excel Macros for all lipid classes according to principles described previously [43]. In brief, data analysis was performed with MassLynx software, which included the NeoLynx tool (Micromass) for averaging the scans at half peak height of the total ion count. NeoLynx generates centroid peak data from the continuum spectra and allows selection of the intensities of certain peaks. Neolynx includes background subtraction and smoothing according to Savitzky Golay of the combined spectra. These NeoLynx results were exported to Excel spreadsheets and further processed by self-programmed Excel macros, which sort the results, correct for isotopic overlap, calculate the ratios to the internal standards, generate calibration curves, and calculate quantitative values [43].

Lipid species were annotated according to the proposal for shorthand notation of lipid structures derived from mass spectrometry [50]. Glycerophospholipid species annotation was based on the assumption of even numbered carbon chains only and presented as the sum of carbon chain length and degree of unsaturation, without specifying fatty acid location at the sn-1 or sn-2 position. SM species annotation was based on the assumption that sphingosine d18:1 is present [50]. Final quantities of lipid species and total lipid (sum of analysed lipid species) were calculated and expressed in nanomoles per milligram wet weight of tissue.

### Statistics

All data are expressed as mean ± standard deviation (SD) with at least three mice (n = 3) for each group. Two-way analysis of variance (ANOVA) was calculated using GraphPad Prism Version 5.4 (GraphPad Software, San Diego, CA). Statistical comparisons among the groups were performed by Bonferroni post-test using the same software. A p-value of 0.05 or lower was considered as significant. Significance is indicated as **** P < 0.0001, *** P < 0.001, ** P < 0.01, *P < 0.05.

## Results

### Overview and analysis of the pulmonary lipidome

**Fig 1:**
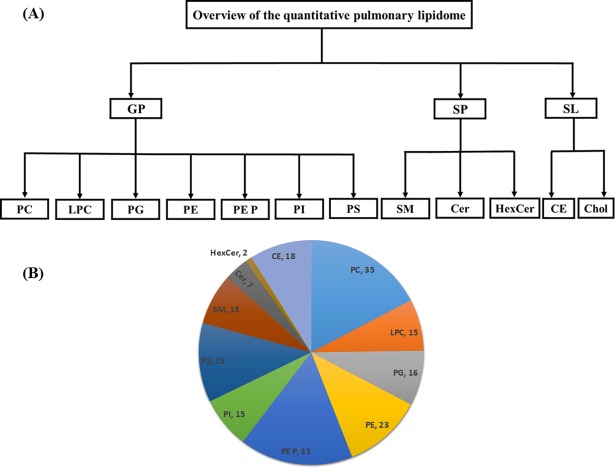
Overview of the quantitative lipidomic analyses of mouse lung homogenates during postnatal development by mass spectrometry. The numbers represent number of lipid species quantified for particular lipid class.

The current study presents an extensive quantitative lipidome analysis of the total mouse lung homogenates during the postnatal development performed with the help of ESI-MS/MS. In total, we performed quantitative analysis of 160 GP, 24 SP, 18 CE species and cholesterol in different stages of the postnatal lung development (lungs of newborn, 15-day-old and 12-week-old adult mice). The glycerophospholipids consist of 35 PC, 15 LPC, 16 PG, 23 PE, 33 PE P, 15 PI and 23 PS lipid species. Sphingolipids consist of 15 SM, 7 Cer and 2 cerebroside (HexCer) lipid species. The overview of total lipid analyses from mouse lung homogenates during development is depicted in Fig 1A and 1B.

### Total lipid quantitation during postnatal lung development

**Fig 2:**
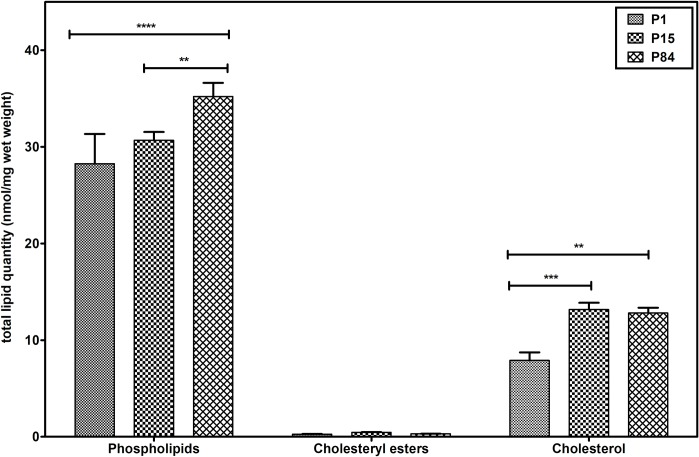
Total lipid quantity of phospholipids, cholesteryl esters and cholesterol in mouse lung during postnatal development. Displayed are nmol/mg wet weight of the lipid class of all analyzed lipid species. Values are represented as mean ± SD, p-value summary: **** P < 0.0001, *** P < 0.001, ** P < 0.01. Where significance is not mentioned, values are considered as being not significant. Phospholipids represents the sum of PC, LPC, PE, PE P, PG, PI, PS, and SM.

To evaluate the total lipid content in mouse lungs during the postnatal development, lipid quantity of phospholipids (sum of all analyzed GP classes), cholesteryl esters and cholesterol was calculated and expressed as nmol/mg wet weight (Fig 2). Total phospholipid content significantly increased during the development from P1 (28.26 ± 3.08 nmol/mg) to P84 (35.20 ± 1.42 nmol/mg) and from P15 (30.68 ± 0.85 nmol/mg) to P84 (35.20 ± 1.42 nmol/mg). However, we did not observe a statistically significant increase between P1 (28.26 ± 3.08 nmol/mg) and P15 (30.68 ± 0.85 nmol/mg). In contrast, cholesterol was significantly increased during progressing from P1 (7.9 ± 0.83 nmol/mg) to P15 (13.16 ± 0.70 nmol/mg) and a significant increase of the values was observed during the development from P1 (7.9 ± 0.83 nmol/mg) to adult lung (12.81 ± 0.55 nmol/mg). However, while developing from P15 to P84, cholesterol remained constant in P84 (12.81 ± 0.55 nmol/mg). The esterified cholesterol species remained at the level of <0.5 nmol/mg wet weight.

Individual lipid class (sum of the analysed lipid species) quantities are presented in S1 Table. For instance, the PC species gradually increased from the stage of P1 (16.47 ± 2.23 nmol/mg) to P84 (18.03 ± 0.70 nmol/mg), but in P15 lung the PC species were lower (14.80 ± 0.57 nmol/mg) as compared to P1 and adult. In contrast, we observed a gradual increase in the total amount of PS, PI, PE, PE P, LPC and SM lipid classes during the postnatal development of the mouse lung. Among these, only PS (3.05 ± 0.19 to 5.35 ± 0.22 nmol/mg) and PE P (2.55 ± 0.23 to 3.82 ± 0.12 nmol/mg) showed statistical significance during the change from P1 to P84.

### Individual lipid species analysis during postnatal lung development

To gain deeper insights into the postnatal developmental alterations, we evaluated the individual lipid species profile of respective lipid classes from mouse lung. The diacylglycerophospholipids were denoted with total number of carbon atoms and double bonds (C:D). LPC and CE species containing single fatty acids were denoted according to lipid species nomenclature.

#### Phosphatidylcholine species

**Fig 3:**
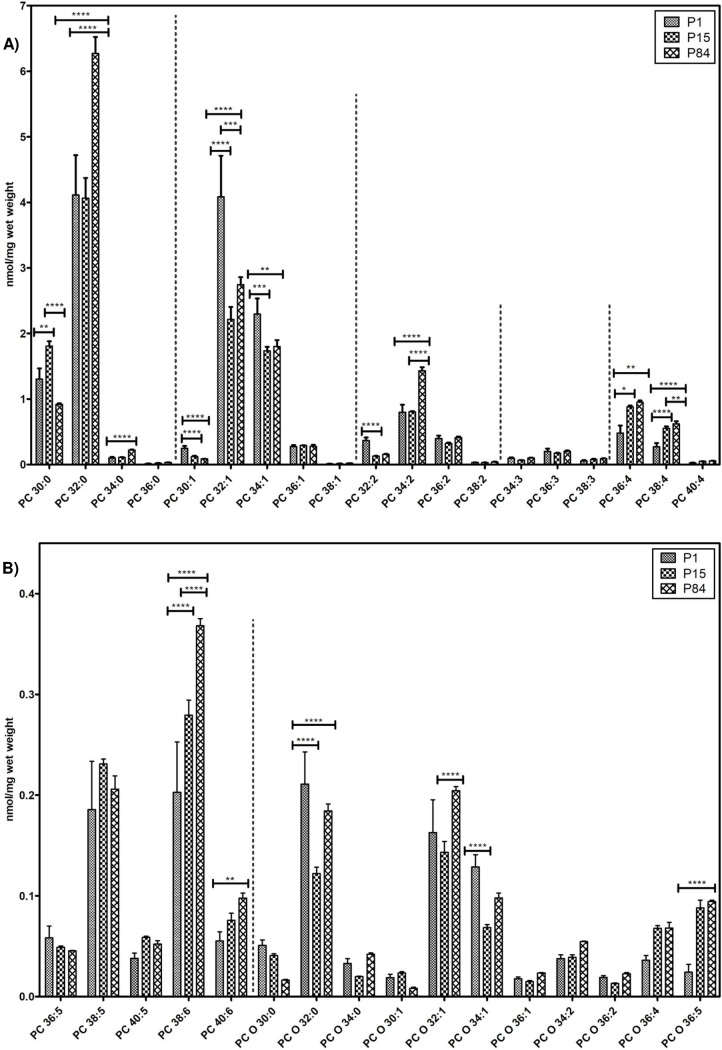
Composition of individual phosphatidylcholine (PC) and ether-phosphatidylcholine (PC O) lipid species during postnatal development of mouse lung. Values are represented as nmol/mg wet weight. A) PC B) PC and PC O. Values are mean ± SD, p-value summary: **** P < 0.0001, *** P < 0.001, ** P < 0.01, *P < 0.05. Where significance is not mentioned, values are considered as being not significant.

In total, 35 different PC (24 PC, 11 PC O) species with different chain length and degree of unsaturation were documented in the lung extracts and their composition is depicted in Fig 3A and 3B. As expected, PC 32:0, PC 32:1, PC 30:0, PC 34:1 and PC 34:2 were the most abundant phosphatidylcholine species in the tested stages in the mouse lung. The predominant PC 32:0 increased significantly while progressing from P1 (4.11 ± 0.6 nmol/mg) to P84 (6.27 ± 0.2 nmol/mg). Similarly, PC 34:2, PC 36:4, PC 38:6, PC 38:4 and PC 40:6 lipid species were significantly elevated in P84 as compared to P1 (Fig 3A & 3B).

In contrast, monounsaturated lipid species (total number of double bonds = 1) PC 34:1 showed a significant reduction during the development from P1 (2.23 ± 0.23 nmol/mg) to P15 (1.73 ± 0.06 nmol/mg) and remained constant in P84 (1.79 ± 0.09 nmol/mg). Similarly, the values of PC 32:1 also significantly dropped during the maturation from P1 (4.08 ± 0.6 nmol/mg) to P15 (2.21 ± 0.19 nmol/mg) and thereafter slightly increased from the stage P15 to P84 (2.74 ± 0.11 nmol/mg). The monounsaturated lipid species (PC 30:1, 32:1, 34:1) were elevated in P1 lungs (Fig 3A).

The PC 30:0 gradually increased from the stage of P1 (1.3 ± 0.1 nmol/mg) to P15 (1.8 ± 0.07 nmol/mg) and then significantly decreased in P84 (0.91 ± 0.01 nmol/mg). Interestingly, higher levels of PC 30:0 were detected in P15 lungs in comparison to P1 and P84.

The analyzed ether-phosphatidylcholine (PC O) lipid species were present at low concentrations and their quantitative information during postnatal pulmonary development is showed in Fig 3B.

#### Lysophosphatidylcholine species

**Fig 4:**
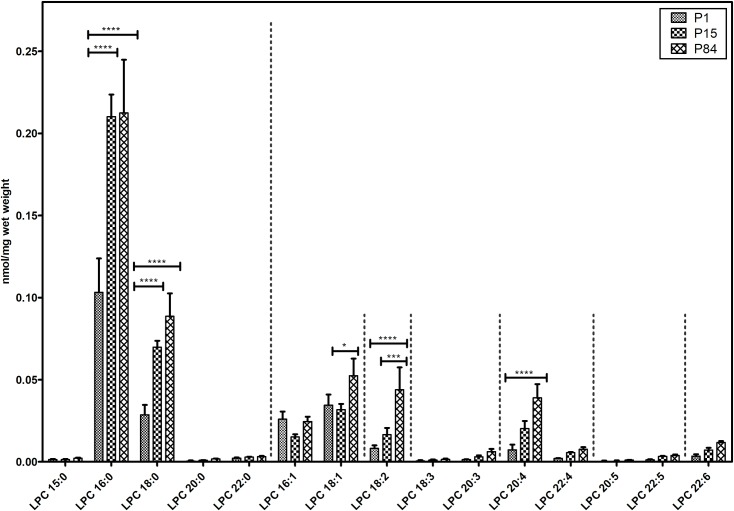
Composition of individual lysophosphatidylcholine (LPC) lipid species during postnatal development of mouse lung. Values are represented as nmol/mg wet weight. Values are mean ± SD, p-value summary: **** P < 0.0001, *** P < 0.001, ** P < 0.01, *P < 0.05. Where significance is not mentioned, values are considered as being not significant.

In total, 15 different lysophosphatidylcholine lipid species were analyzed and their composition pattern is depicted in Fig 4. The major LPC species detected were LPC 16:0 followed by 18:0, 18:1, 16:1, 18:2 and 20:4. The values of LPC 16:0 significantly increased during the maturation from P1 (0.103 ± 0.02 nmol/mg) to P15 (0.210 ± 0.01 nmol/mg) and P1 to P84 (0.212 ± 0.03 nmol/mg). Similarly, during development process from newborn to adult the amounts of all other LPC species including LPC 18:0, polyunsaturated LPC species 18:2 and 20:4 increased. Remaining LPC species occurred at lower concentrations and did not show any significant differences.

#### Phosphatidylglycerol species

**Fig 5:**
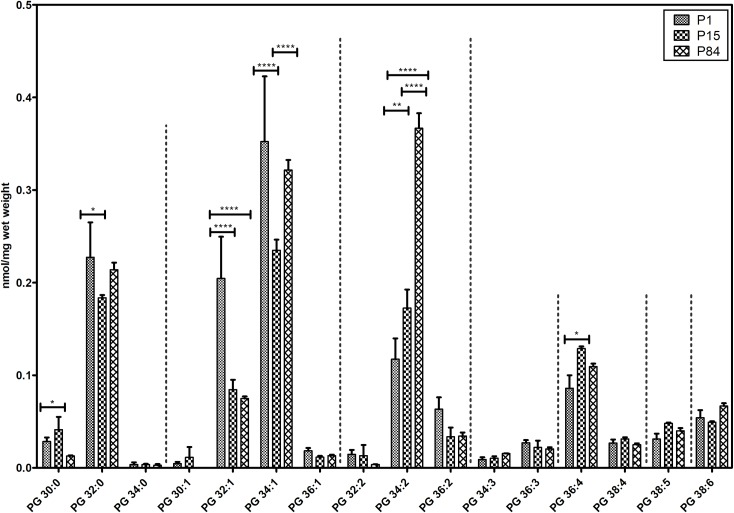
Composition of individual phosphatidylglycerol (PG) lipid species during postnatal development of mouse lung. Values are represented as nmol/mg wet weight. Values are mean ± SD, p-value summary: **** P < 0.0001, *** P < 0.001, ** P < 0.01, *P < 0.05. Where significance is not mentioned, values are considered as being not significant.

We analyzed 16 individual lipid species of phosphatidylglycerol and their composition during the postnatal lung development is represented in Fig 5. As expected, PG 34:1, PG 34:2, PG 32:0 and PG 32:1 constituted highly abundant lipid species that were detected in all three groups. Interestingly, in contrast to PC 32:0, disaturated PG 32:0 decreased while progressing from P1 to P15. Notably, monounsaturated PG 32:1 significantly dropped in P84 (0.075 ± 0.002 nmol/mg) as compared to P1 (0.205 ± 0.04 nmol/mg). Similarly, the values of PG 34:1 dropped in P15 as compared to P1 and slightly increased while maturing from P15 to P84.

In contrast, diunsaturated (total number of double bonds = 2) PG 34:2 shows a significant increase during the development from P1 (0.117 ± 0.023 nmol/mg)) to P15 (0.173 ± 0.020 nmol/mg) and from P15 to P84 (0.367 ± 0.016 nmol/mg).

Similar to PC 30:0, the PG 30:0 was higher in P15 in comparison to P1 and P84. Interestingly, we detected higher levels of PG 36:4 in P15 lungs. Remaining PG lipid species did not show any statistically significant differences during development.

#### Phosphatidylethanolamine species

**Fig 6:**
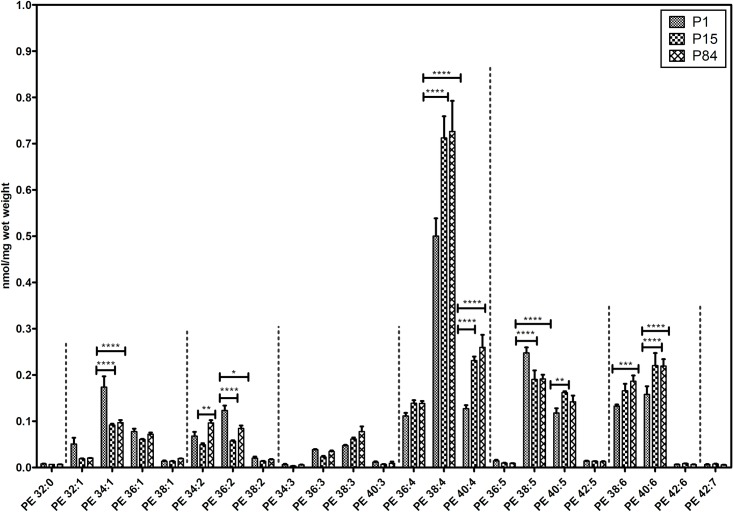
Composition of individual phosphatidylethanolamine (PE) lipid species during postnatal development of mouse lung. Values are represented as nmol/mg wet weight. Values are mean ± SD, p-value summary: **** P < 0.0001, *** P < 0.001, ** P < 0.01, *P < 0.05. Where significance is not mentioned, values are considered as being not significant.

Total 23 individual phosphatidylethanolamine lipid species were quantified and their composition is depicted in Fig 6. Interestingly, in comparison to the other lipid classes, PE lipids exhibited higher abundance of long chain polyunsaturated species. Strikingly, PE 38:4 showed higher abundance in all three groups in comparison to other PE species. Further, PE 38.4 significantly increased during maturation from P1 (0.50 ± 0.038 nmol/mg) to P15 (0.712 ± 0.046 nmol/mg) and remained constant in P84 (0.726 ± 0.066 nmol/mg). Similarly, PE 40:4, PE 40:5, PE 40:6 and PE 38:6 were relatively abundant in P84 and significantly increased during the development of the lung. In contrast, less abundant PE 34:1, 36:2 and 38:5 exhibited higher concentrations in P1 but then significantly decreased in P84. The concentration of other lipid species were low abundance and it did not show any significant differences in all three groups.

#### PE based plasmalogens

**Fig 7:**
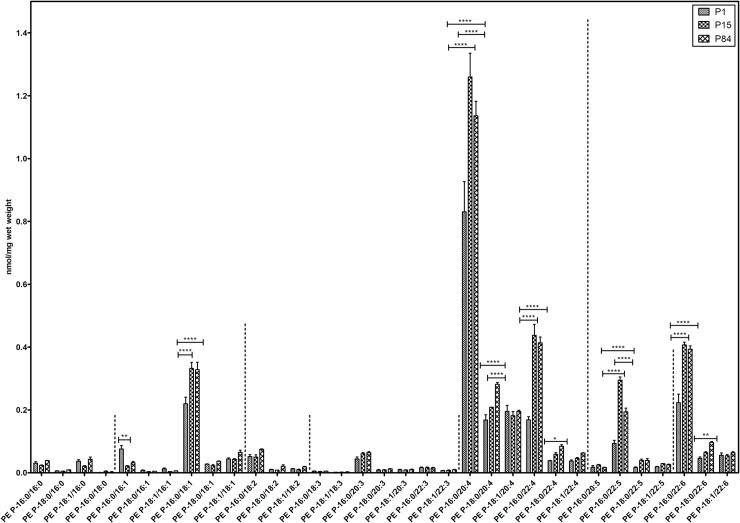
Composition of individual PE based plasmalogen (PE P) lipid species during postnatal development of mouse lung. Values are represented as nmol/mg wet weight. Values are mean ± SD, p-value summary: **** P < 0.0001, *** P < 0.001, ** P < 0.01, *P < 0.05. Where significance is not mentioned, values are considered as being not significant.

PE P-16:0, PE P-18:0 and PE P-18:1 (sn-1) individual plasmalogen compositions were calculated and their values have been displayed in Fig 7. Interestingly, PE P-16:0 (sn-1 substituent) plasmalogens are present in higher amounts in all three groups. Regardless of the alkenyl chain in sn-1, the plasmalogens containing PUFAs in sn-2 position were the most abundant, represented mainly by 20:4 followed by 22:6, 22:4 and 22:5 in all tested groups.

The PE P-16:0/22:4 and PE P-16:0/22:6 significantly rose during the postnatal lung development (from P1 to P15 and P1 to P84). In contrast, the predominant PE P-16:0/20:4 and PE P-16:0/22:5 also rose during development from P1 to P15, however, a slight decrease from P15 to P84 was noted. Similarly, PE P-18:0 based 20:4, 22:4, 22:6 and PE P-16:0/18:1 lipid species increased significantly during the development process (P1 to P84). However, the values of low abundant PE P-16:0/16:1 were significantly lower in P84 as compared to P1. Other individual ethanolamine based plasmalogens were not significant during the postnatal lung development.

#### Phosphatidylinositol and phosphatidylserine species

**Fig 8:**
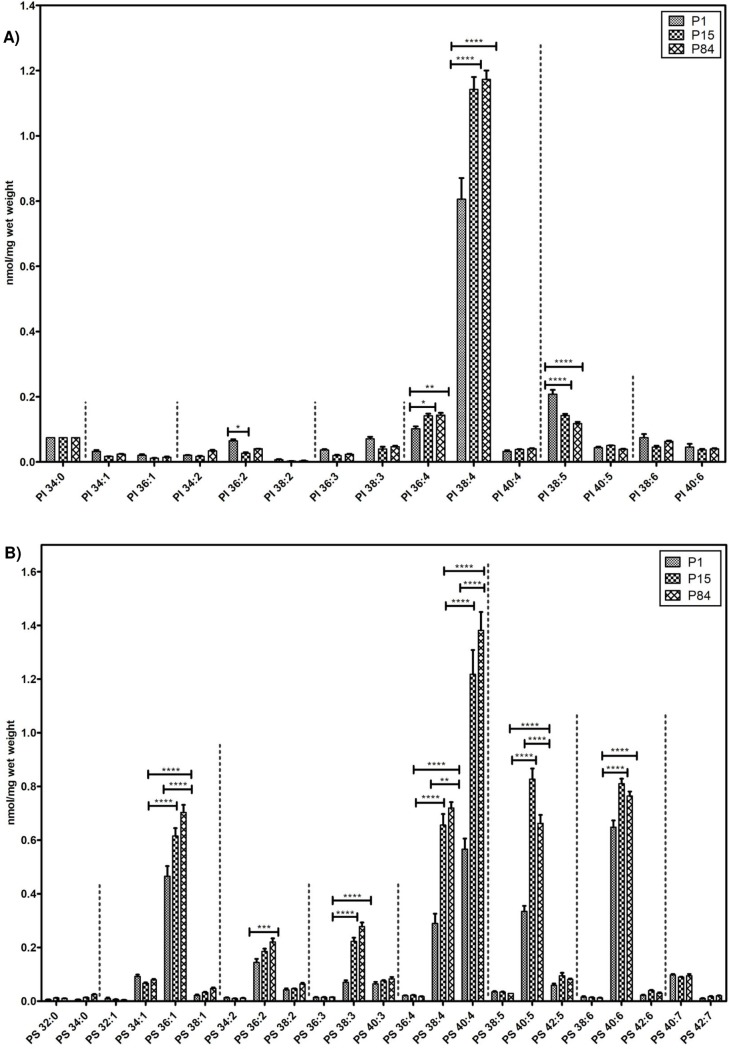
Composition of individual phosphatidylinositol (PI) and phosphatidylserine (PS) lipid species during postnatal development of mouse lung. Values are represented as nmol/mg wet weight. A) PI B) PS. Values are mean ± SD, p-value summary: **** P < 0.0001, *** P < 0.001, ** P < 0.01, *P < 0.05. Where significance is not mentioned, values are considered as being not significant.

The individual composition of 15 species of phosphatidylinositol and 23 species of phosphatidylserine is depicted in Fig 8A and 8B respectively. Similarly, to PE and PE based plasmalogens, both PI and PS were found to be highly enriched with polyunsaturated species. PI 38:4 was the most abundant during all developmental stages. PI 38:4 (0.806 ± 0.065 nmol/mg to 1.173 ± 0.027 nmol/mg), PI 36:4 were significantly increasing while progressing from P1 to P84, whereas less abundant PI 36:2 and PI 38:5 species were higher in newborn and significantly gradually decreasing during the postnatal lung development (Fig 8A).

Among PS, polyunsaturated species (total number of double bonds ˃2) 40:4, 40:5, 40:6, 38:4 and the PS 36:1 were highly abundant in all stages of lung development. PS 40:4 was significantly gradually increasing from the phase of P1 (0.566 ± 0.04 nmol/mg) to P15 (1.218 ± 0.09) and from P15 to P84 (1.381 ± 0.06 nmol/mg). PS 38:4 and 36:1 species followed the same pattern (rise from P1 to P15 and P15 to P84). In addition, PS 36:2, 38:3 and 40:6 was detected in P1 and their increase was observed in P84. Among the PS species, PS 40:5 exhibited higher levels in P15 (0.827 ± 0.039 nmol/mg) as compared to P1 (0.335 ± 0.021 nmol/mg) and P84 (0.662 ± 0.031nmol/mg). The other analyzed PS species did not reach significance during lung development (Fig 8B).

#### Sphingomyelin and ceramide species

**Fig 9:**
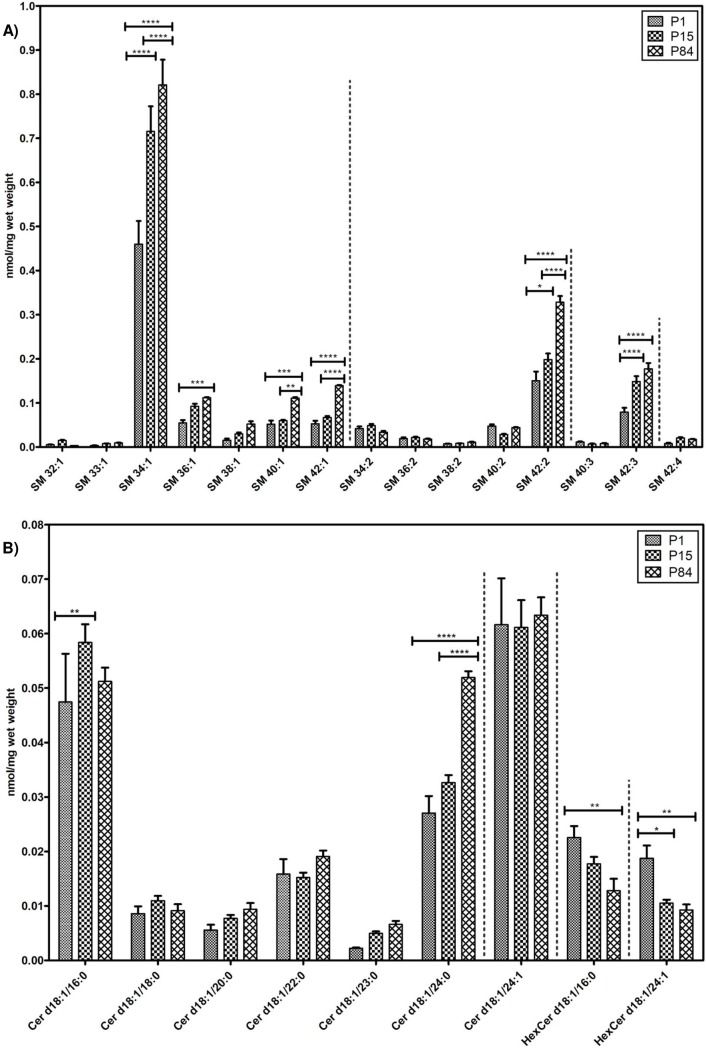
Composition of individual sphingomyelin (SM) and ceramide (Cer) lipid species during postnatal development of mouse lung. Data are represented as nmol/mg wet weight. A) SM B) Cer. Values are mean ± SD, p-value summary: **** P < 0.0001, *** P < 0.001, ** P < 0.01, *P < 0.05. Where significance is not mentioned, values are considered as being not significant.

Within sphingolipids, individual 15 sphingomyelin, 7 ceramide and 2 cerebroside species compositions are depicted in Fig 9A and 9B respectively. SM 34:1 was the most dominant SM species in all developmental stages and gradually, but significantly increasing from the phase of P1 (0.46 ± 0.052 nmol/mg) to P15 (0.716 ± 0.05 nmol/mg), and thereafter slightly increasing from P15 to P84 (0.821 ± 0.057 nmol/mg). Similarly, SM 42:2 followed the same pattern during development.

The very long chain SM lipid species 40:1, 42:1and 42:3 were significantly rising during the postnatal lung development and their elevated levels were detected in P84 (Fig 9A).

Cer d18:1/16:0 was present in higher amounts in P15 lungs in comparison to P1 and P84. The very long chain fatty acid containing ceramide lipid species Cer d18:1/24:0 exhibited higher levels in P84. Interestingly, both of the analyzed HexCer d18:1/16:0 and HexCer d18:1/24:1 was present at higher levels in P1 and a significant gradual decrease was observed during the postnatal lung development up to the stage of P84 (Fig 9B).

#### Cholesteryl esters

**Fig 10:**
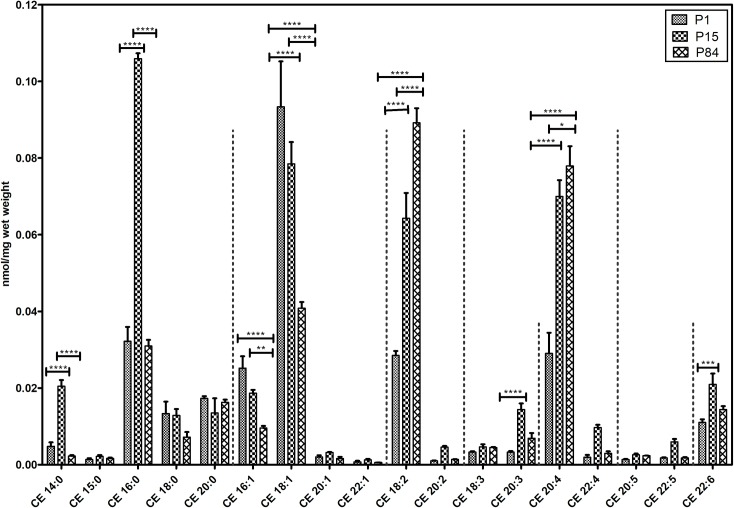
Composition of individual cholesteryl ester (CE) lipid species during postnatal development of mouse lung. Data are represented as nmol/mg wet weight. Values are mean ± SD, p-value summary: **** P < 0.0001, *** P < 0.001, ** P < 0.01, *P < 0.05. Where significance is not mentioned, values are considered as being not significant.

Eighteen esterified forms of cholesterol were analysed and their composition is depicted in Fig 10. Similar to LPC, the myristic acid (14:0) and palmitic acid (16:0) containing CE species were significantly elevated in the phase of P15 in comparison to P1 and P84. Oleic (18:1), palmitoleic (16:1) containing CE species were more elevated in P1 and significantly lowered during development. In contrast, linoleic (18:2) and arachidonic (20:4) acid containing CE species were significantly gradually increasing from the stage of P1 and reached higher levels in P84.

Furthermore, the distribution pattern of GP according to their carbon chain length (total number of carbon atoms), degree of unsaturation (total number of double bonds) was elaborated and the results are shown in S2 Table, S1 Fig. Overall, the PC and PG comprised higher amounts of lipid species with carbon chain length ≤36. Whereas PE, PS and PI contained higher amount of lipid species with carbon chain length ˃36.

## Discussion

Lipids constitute a diverse group of biomolecules, playing many roles in lung biology, especially in reducing the surface tension of alveoli to prevent the alveolar collapse and thereby stabilizing the lung parenchyma. The lung major lipid classes originating either from the pulmonary surfactant or from the bronchoalveolar lavage fluid (BALF) were already partly characterized, but a detailed distribution of total lipid classes and of their individual lipid molecular species composition in mouse lung during its postnatal development however, are not fully understood. In our current study, we used direct flow injection electrospray ionization tandem mass spectrometry to provide the total lipid quantity, and significant stage specific alterations of individual lipid species during the process of mouse postnatal pulmonary development. Furthermore, we showed the distribution pattern of lipid classes according to their carbon chain length and degree of unsaturation during development process.

In the present study, we analyzed and quantified total 202 lipid species (GP, SP, and CE) and cholesterol of the pulmonary lipidome from the mouse lung homogenates of the P1, P15 and adult lungs (Fig 1A & 1B and Fig 2 & S1 Table). In general, an increase in the total lipid quantity (nmol/mg wet weight) of pulmonary tissue is a characteristic change during the lung development process. Our results revealed increased levels of the total phospholipid and cholesterol content during development. Indeed, these results are in consistence with the findings of Williams and colleagues as well as of Hahn, et al. in which rat lung and other organ lipids were studied during maturation [51, 52].

Furthermore, pulmonary lipid distribution patterns of the whole lung tissue in comparison to the pulmonary surfactant lipid composition in different mammalian species were studied (see book chapters in ref) [3, 53]. In this context, it is important to mention that the distribution pattern of the lipids in the lung tissue is heterogeneous between distinct species. In general, PC’s were the most predominant class of lipids of all tested species [32]. The second most abundant lipid class of the surfactant GP was PG, however, this lipid class was detected in lower amounts in the whole lung tissue. In contrast, PE represented the minor class of lipids in the surfactant but constituted second major lipid class following PC in the whole lung tissue. Similarly, SM and PS lipid classes were detected in lower amounts in the pulmonary surfactant, however, they also were described as being abundant in the whole lung tissue. In fact, our results of the lipid class distribution of the mouse lung homogenates are in agreement with these findings [3, 53]. Our data clearly indicate that PC and cholesterol occupy major parts of the whole lung lipidome (Panel A in S2 Fig). Among the phospholipids, PC is the most dominant lipid class followed by PE (PE+PE P), PS, SM, PI and PG present during the postnatal mouse lung development (Panel B in S2 Fig). Based on these composition values, we calculated the molar ratios of lipid classes (S3 Table). For instance, the PC/LPC ratio is decreased from P1 to P15, and from P15 to P84. In contrast, SM/Cer molar ratio significantly increased from P1 to P15 and from P15 to P84.

Interestingly, we observed a significant increase in the concentration of cholesterol from the phase of newborn to P15 (Fig 2, S1 Table & Panel A in S2 Fig), suggesting that cholesterol may play an important role in the process of alveolarization. Cholesterol is an integral component of various cell membranes, involved in the maintenance of membrane fluidity, membrane functions and signal transduction. In fact, cholesterol is the major neutral lipid component of the lung and up to 80% of the cholesterol present in the lung is in surfactant [54], and it is considered as a protosurfactant in immature lungs, lungs with lack of septation and in saccular lungs [55]. Moreover, cholesterol enhances the adsorption of DPPC by increasing membrane fluidity and control the surface viscosity of the surfactant [56, 57].

In our study, we focused on the alterations of individual lipid species of mouse lungs during the postnatal development. Our results showed a significant increase in the abundance of PC 30:0 during the alveolarization (P1 to P15) (Fig 3A) process. These results are supported by previous observations on the postnatal development of the lung tissue and surfactant lipid analyses from 8-day-old mice and adult animals [41]. Bernhard and co-workers reported about significant alterations in the abundance of major PC lipid molecular species in the pulmonary surfactant of different mammalian species during lung development [31, 40, 41].

In contrast to PC findings of surfactant lipidome, we showed a significant gradual increase of PC 32:0 (most likely to be DPPC) during the postnatal lung development. DPPC is the primary surface-active material found in majority of the mammalian species pulmonary surfactant. Maintenance of adequate DPPC within air space is essential for normal lung function [58]. RDS is the major cause of mortality and morbidity in premature infants diagnosed with mainly DPPC deficiency in quantity and quality of pulmonary surfactant [59]. Currently, surfactant replacement therapy with added products of DPPC is an effective therapeutic strategy available for RDS management [60]. Interestingly, high contents of monounsaturated lipids of PC 32:1 and PC 34:1 was detected in P1 mice in comparison to P15 and adult lungs, suggesting that PC 32:1 might be involved in the establishment of the air-liquid interface in newborn animals. Furthermore, PC 34:1 is known to be crucial and plays an important role in the adsorption of DPPC immediately after birth [61, 62]. In contrast to mice, PC 30:0 is completely absent and PC 32:1 is minimal in nonalveolar species (birds), in which the lung contains capillaries instead of alveoli, suggesting that, PC 30:0 and PC 32:1 species are important and play an active dynamic role in the alveolarization process [30]. In this context, it is important to mention the recent findings that PC 30:0 inhibited macrophage-triggered proliferation of T-lymphocytes and decreased the production of reactive oxygen species (ROS) during alveolarization [63, 64]. Moreover, PC 30:0 was significantly reduced in the emphysema patients and infants with BPD as well as in the neonatal rat models of reduced alveolarization suggesting that PC 30:0 may serve as a diagnostic marker for alveolar size during diseases [65]. Similarly to these observations, in the pig model, PC 30:0 was found high in abundance in newborns and gradually decreased with age in adolescent pigs [31], whereas in humans and guinea pigs, PC 30:0 was increased during the lung development [41]. The specific functions of PC 30:0, PC 32:1 and PC 34:1 lipid species during postnatal lung development are not clear yet. Also, the molecular mechanism and alterations of lipid species during the lung development are not clear yet. These alterations may be specific for individual mammalian species, a supposition that requires further investigation.

Phosphatidylglycerol lipid concentrations are highly concentrated in the lung compared to other mammalian tissues [66]. It is well documented that PG lipids are involved in the adsorption and, spreading of surfactant over the epithelial surface, as well as influence innate immunity and protect against viral infections [9, 67, 68]. Interestingly, surfactant deficiency in premature infants and also in mouse models of BPD showed the complete absence of PG lipids [69]. Indeed, the presence of PG lipids in amniotic fluid is an indicator for the fetal lung maturity and PG lipids are known to be vital in the management of neonatal RDS and other obstetric conditions [70]. It is known that PG lipid species are crucial for the lung function. There are, however, no reports on the composition of PG 30:0 during the postnatal lung development. In fact, Bernhard and colleagues were not able to measure PG 30:0 from rat surfactant during the postnatal lung development [40]. Remarkably, in our study, we observed a significant increase in PG 30:0 abundance during alveolarization (P1 to P15), similar to PC 30:0, probably because of the high content of myristic acid during the postnatal lung development. In contrast to DPPC, we observed a significant decrease of abundance of DPPG during postnatal lung development. Recent findings suggest that DPPG interacts with vaccinia and variola virus strains and reduces the infection of pneumocytes in respiratory poxvirus infection [68]. Moreover, several reports demonstrated that PG 34:1 (palmitoyl-oleoyl-phosphatidylglycerol, POPG) acts as a potent antiviral lipid against influenza A and respiratory syncytial virus [9, 67]. Interestingly, we observed a high content of this antiviral lipid PG 34:1 (POPG) in the newborn mouse lungs in comparison to P15 and P84 (Fig 5), suggesting that they might improve the innate immunity against viruses during the perinatal period.

In addition to PC and PG, we measured other lipid species in the lung tissue such as PE, PI and PS (Figs 6, 8A & 8B). In contrast to PC and PG, we found that these lipid classes were found to be mostly enriched with long chain polyunsaturated species. Monounsaturated lipid species (PE 32:1) were found in high abundance in P1 in comparison to P15 and P84. In fact, long chain polyunsaturated species serve as substrates for the pro-inflammatory (leukotrienes, prostaglandins, etc.), as well as anti-inflammatory and pro-resolution (lipoxins, etc.) lipid mediators [71]. Furthermore, mass spectrometry imaging of adult mouse lungs showed that these long chain polyunsaturated lipids are highly abundant at the epithelial lining of airways [72]. We observed that 38:4 lipids were abundant in case of PE and PI lipid classes in all three age groups, which may serve as a source for arachidonic acid (AA) for the generation of lipid mediators. Proteomics data of a recent study revealed that proteins (cyclooxygenases, lipoxygenases, etc.) responsible for the generation of bioactive lipid mediators, are significantly upregulated in the adult mouse lungs, suggesting that these long chain polyunsaturated lipid species serve as a source for AA [27].

Quantitative information of less abundant species of these lipid classes would help to understand postnatal developmental alterations in detail. However, the mechanism of alterations of individual lipid species of these lipid classes during lung development needs to be further explored.

Plasmalogens are glycerophospholipids characterized by a vinyl ether linkage in sn-1 and an ester linkage in sn-2 position of the glycerol backbone. Plasmalogens are involved in the membrane dynamics, serve as an endogenous antioxidants, protect against ROS and prevent lipoprotein oxidation [73]. Plasmalogen biosynthesis starts in the peroxisomes and deficiency of plasmalogens is associated with various peroxisomal disorders [74] and other respiratory diseases like BPD [75], asthma and COPD [76]. We observed that PE-based plasmalogens are much higher abundant compared to ether-phosphatidylcholines (PC O) during the postnatal mouse lung development. In ethanolamine plasmalogens, PE P-16:0 plasmalogens comprised the highest amount, whereas, PE P-18:0 and PE P-18:1 made up a smaller amount (Fig 7). Interestingly, we observed a high content of 20:4, 22:6, 22:5 and 22:4 (most likely to be arachidonic acid AA, docosahexaenoic acid DHA, docosapentaenoic acid DPA and adrenic acid)-rich plasmalogens (Fig 7). Further, the total quantities of plasmalogens (sum of all analyzed PE P species) were gradually increased from P1 to P84 during the postnatal lung development (S1 Table). These results are in consistent with the previous study, in which high abundance of PE P species were noted in adult mouse lungs during postnatal pulmonary developmental processes [27]. Arachidonic acid enriched plasmalogens seem to play an important role in immune defence and normal lung physiology [73]. Plasmalogens are reported to serve as a reservoir for the precursor molecules (e.g., AA, EPA, DHA, and DPA etc.) of eicosanoids, which are biologically active secondary lipid signalling messengers or for maresin and resolvins, lipid derivatives involved in the regulation of inflammation [77, 78]. Rüdiger and colleagues showed that addition of plasmalogens to surfactant-like phospholipid mixtures reduces surface tension [79] and high content of plasmalogens in tracheal aspirate of preterm infants reduces the risk of respiratory diseases [80]. Likewise, another study reported that high contents of plasmalogens protects the endothelial cells from hypoxia and ROS mediated stress [81].

Sphingolipids are primarily found in cell membranes and are involved in diverse biologic processes such as migration, proliferation, differentiation, senescence, cell death, autophagy, and efferocytosis [82]. In the lungs, sphingolipids are associated with cystic fibrosis, asthma, pulmonary edema, BPD, inflammation, lung injury and various types of lung cancers [83]. Ceramides show both proliferative and apoptotic effects depending on their concentration and chain length [84]. In analyzed SM lipid species, we detected that SM 34:1 lipid species as being predominant in all stages (Fig 9A). Both sphingomyelin 34:1 and ceramide species (Cer d18:1/16:0) showed high contents during alveolarization, especially in P15 (Fig 9A & 9B) mouse lungs, suggesting that, these lipid species are involved in the remodelling of tissue, also observed in the rat lung development [85]. In contrast to our findings (P1 to P15), a recent study on mouse lungs using LC-MS/MS approach showed no significant alterations in the Cer d18:1/16:0 levels from P7 to P14 [27]. The total content of sphingomyelin gradually increased with the age and the necessary transfer of biochemical substances across the semipermeable membranes (S1 Table).

So far, no reports are available about the developmental changes of less abundant cholesteryl ester species in the mouse lung. Saturated fatty acids such as myristic acid (14:0)- and, palmitic acid (16:0)-containing lysophosphatidylcholines (Fig 4) and cholesteryl esters (Fig 10) were found highly abundant during P15 in comparison to P1 and P84. Monounsaturated fatty acid (MUFA) containing CE species were elevated in the newborn, whereas polyunsaturated fatty acid (PUFA) containing CE species were elevated in adult lungs. Physicochemical properties of lipids depend on their chain length and their degree of unsaturation. In this aspect, we calculated the distribution patterns of glycerophospholipids according to their carbon chain length (number of carbon atoms). We observed that, PC and PG glycerophospholipids are highly abundant of lipid species with carbon chain length C≤36, whereas PE, PS and PI glycerophospholipids are highly abundant with long chain lipid species (C˃36). The majority of the monounsaturated glycerophospholipids were found to be highly abundant in newborns, whereas polyunsaturated lipid species were highly abundant in adult lungs (S1 Fig). In contrary, P15 lungs exhibited high contents of myristic (14:0)- and palmitic (16:0)-acid containing lipid species.

## Limitations and weaknesses of the study

In the current study, we performed an extensive quantitative lipidomic analysis of P1, P15 and P84 mouse whole lung tissue homogenates to understand the changes occurring during postnatal development. The data provides lipidomic alterations in mouse lung during developmental process. However, at this stage we are not able to discriminate the lipidomic changes occurring specifically at cellular (membrane or intracellular) and extracellular (alveolar) level. Comprehensive comparative (quantitative) lipidomic analysis of bronchoalveolar lavage fluid (BALF) and whole lung tissue homogenates in mice and other mammalian species in which alveolarization continues beyond extra-uterine life (e.g. rats) needs to be investigated in the near future, which can provide deeper insights for a better understanding of pulmonary developmental process at molecular and cellular level.

## Conclusion

In our study, we have provided the total lipid quantity and given a detailed overview of lipid classes as well as absolute quantitative information on the individual lipid species and their distribution pattern according to carbon chain length and degree of unsaturation during postnatal mouse lung development using high-throughput tandem mass spectrometry. Our study provides an extensive quantitative lipidome of whole mouse lung tissue (including less abundant lipid species, neutral lipid components such as cholesterol and their esters), which may serve as reference for understanding the occurring lipid alterations, which in turn affect lung function during development or in pulmonary diseases.

## Supporting information

S1 Table Quantitative analyses of individual lipid classes in mouse lung during postnatal development. Values are expressed as nmol/mg wet weight and represented as mean ± SD.
(DOC)

S2 Table Distribution of glycerophospholipids according to carbon chain length (total number of carbon atoms) and alkenyl chain (PE P) during postnatal development of mouse lung. Values are represented as nmol/mg wet weight.
(DOC)

S3 Table Molar ratios of lipid classes. Displayed are the values of molar ratios of lipid classes. Values are represented as mean ± SD.
(DOC)

S1 Fig Distribution of glycerophospholipids according to degree of unsaturation (total number of double bonds) during postnatal development of mouse lung. Saturated (total number of double bonds = 0), Monounsaturated (total number of double bonds = 1), Polyunsaturated (total number of double bonds ≥2) lipids.
(TIF)

S2 Fig Total lipid composition of mouse lung during postnatal development. The displayed values are mol% of the respective lipid class of all analyzed lipids. Panel A) Glycerophospholipids (GP), sphingolipids (SP), cholesteryl esters (CE) and cholesterol. Panel B) only GP, SP without CE and cholesterol. Values are represented as mean ± SD, p-value summary: **** P < 0.0001, *** P < 0.001, ** P < 0.01, *P < 0.05.
(TIF)

S1 File Lipid profiling of mouse lung during postnatal development used in this study. The list of identified and quantified lipid molecules, experimental protocol, abbreviations and statistics.
(XLS)

## Abbreviations

PC: phosphatidylcholine
LPC: lysophosphatidylcholine
PC O: ether-phosphatidylcholine
PG: phosphatidylglycerol
PE: phosphatidylethanolamine
PE P: PE based plasmalogen
PI: phosphatidylinositol
PS: phosphatidylserine
SM: sphingomyelin
Cer: ceramide
HexCer: hexosylceramide
GP: glycerophospholipids
SP: sphingolipids
SL: sterol lipids
CE: cholesteryl esters
ESI: electrospray ionization
MS/MS: tandem mass spectrometry
P1: newborn mice
P15: 15-day-old mice
P84: 12-week-old adult mice
