# Supplementary material for: Quantitative lipidomic analysis of mouse lung during postnatal development by electrospray ionization tandem mass spectrometry
Source: PLoS One. 2018 Sep 7;13(9):e0203464. doi: 10.1371/journal.pone.0203464 (PMC6128551; doi:10.1371/journal.pone.0203464)
Supplement: S1 Table — Values are expressed as nmol/mg wet weight and represented as mean ± SD. (DOC) [file pone.0203464.s001.doc]

| **Lipid** | **P1** | **P15** | **P84** |
| --- | --- | --- | --- |
| PC | 16.47 ± 2.23 | 14.80 ± 0.57 | 18.03 ± 0.70 |
| LPC | 0.22 ± 0.05 | 0.39 ± 0.03 | 0.50 ± 0.09 |
| PE | 2.08 ± 0.09 | 2.27 ± 0.12 | 2.44 ± 0.19 |
| PE P | 2.55 ± 0.23 | 3.77 ± 0.18 | 3.82 ± 0.12 |
| PG | 1.27 ± 0.21 | 1.08 ± 0.10 | 1.32 ± 0.03 |
| PI | 1.58 ± 0.12 | 1.74 ± 0.06 | 1.81 ± 0.04 |
| PS | 3.05 ± 0.19 | 5.12 ± 0.26 | 5.35 ± 0.22 |
| SM | 1.05 ± 0.12 | 1.51 ± 0.11 | 1.94 ± 0.09 |
| Phospholipids | 28.26 ± 3.08 | 30.68 ± 0.85 | 35.20 ± 1.42 |
| Cer | 0.17 ± 0.02 | 0.19 ± 0.01 | 0.21 ± 0.01 |
| HexCer | 0.041 ± 0.004 | 0.028 ± 0.002 | 0.022 ± 0.003 |
| CE | 0.27 ± 0.02 | 0.45 ± 0.02 | 0.31 ± 0.00 |
| Cholesterol | 7.90 ± 0.83 | 13.16 ± 0.70 | 12.81 ± 0.55 |
