# Supplementary material for: Quantitative lipidomic analysis of mouse lung during postnatal development by electrospray ionization tandem mass spectrometry
Source: PLoS One. 2018 Sep 7;13(9):e0203464. doi: 10.1371/journal.pone.0203464 (PMC6128551; doi:10.1371/journal.pone.0203464)
Supplement: S2 Table — Values are represented as nmol/mg wet weight. (DOC) [file pone.0203464.s002.doc]

**PC:**

| **Postnatal stage** | **≤34** | **˃34** | **≤36** | **˃36** |
| --- | --- | --- | --- | --- |
| P1 | 13.41 | 2.31 | 14.84 | 0.88 |
| P15 | 11.04 | 3.11 | 12.78 | 1.37 |
| P84 | 13.73 | 3.47 | 15.65 | 1.55 |

**PG:**

| **Postnatal stage** | **≤34** | **˃34** | **≤36** | **˃36** |
| --- | --- | --- | --- | --- |
| P1 | 0.96 | 0.30 | 1.15 | 0.11 |
| P15 | 0.75 | 0.32 | 0.95 | 0.12 |
| P84 | 1.01 | 0.30 | 1.18 | 0.13 |

**PE:**

| **Postnatal stage** | **≤34** | **˃34** | **≤36** | **˃36** |
| --- | --- | --- | --- | --- |
| P1 | 0.31 | 1.77 | 0.67 | 1.40 |
| P15 | 0.17 | 2.10 | 0.45 | 1.80 |
| P84 | 0.23 | 2.21 | 0.56 | 1.87 |

**PS:**

| **Postnatal stage** | **≤34** | **˃34** | **≤36** | **˃36** |
| --- | --- | --- | --- | --- |
| P1 | 0.13 | 2.91 | 0.77 | 2.27 |
| P15 | 0.11 | 5.01 | 0.94 | 4.17 |
| P84 | 0.13 | 5.21 | 1.08 | 4.26 |

**PI:**

| **Postnatal stage** | **≤34** | **˃34** | **≤36** | **˃36** |
| --- | --- | --- | --- | --- |
| P1 | 0.13 | 1.51 | 0.35 | 1.29 |
| P15 | 0.11 | 1.70 | 0.31 | 1.50 |
| P84 | 0.13 | 1.74 | 0.35 | 1.52 |

**PE P:**

| **Postnatal stage** | **P 16:0** | **P 18:0** | **P 18:1** |
| --- | --- | --- | --- |
| P1 | 1.77 | 0.32 | 0.43 |
| P15 | 2.93 | 0.41 | 0.40 |
| P84 | 2.71 | 0.58 | 0.50 |
