# Supplementary material for: Quantitative lipidomic analysis of mouse lung during postnatal development by electrospray ionization tandem mass spectrometry
Source: PLoS One. 2018 Sep 7;13(9):e0203464. doi: 10.1371/journal.pone.0203464 (PMC6128551; doi:10.1371/journal.pone.0203464)
Supplement: S3 Table — Displayed are the values of molar ratios of lipid classes. Values are represented as mean ± SD. (DOC) [file pone.0203464.s003.doc]

| **Molar ratio** | **P1** | **P15** | **P84** |
| --- | --- | --- | --- |
| PC/LPC | 75.43 ± 6.59 | 38.17 ± 3.71 | 36.63 ± 5.37 |
| PC/PE | 7.89 ± 0.80 | 6.54 ± 0.55 | 7.40 ± 0.29 |
| PC/PS | 5.39 ± 0.43 | 2.89 ± 0.04 | 3.37 ± 0.05 |
| PC/PI | 10.42 ± 0.70 | 8.51 ± 0.56 | 9.95 ± 0.20 |
| PE/PS | 0.69 ± 0.02 | 0.44 ± 0.04 | 0.45 ± 0.02 |
| PC/Cholesterol | 2.08 ± 0.07 | 1.13 ± 0.02 | 1.41 ± 0.02 |
| PC/total cholesterol | 2.01 ± 0.08 | 1.09 ± 0.02 | 1.37 ± 0.01 |
| SM/Cer | 6.25 ± 0.26 | 7.93 ± 0.81 | 9.21 ± 0.76 |
