# Supplementary figures and images for: Quantitative lipidomic analysis of mouse lung during postnatal development by electrospray ionization tandem mass spectrometry
Source: PLoS One. 2018 Sep 7;13(9):e0203464. doi: 10.1371/journal.pone.0203464 (PMC6128551; doi:10.1371/journal.pone.0203464)

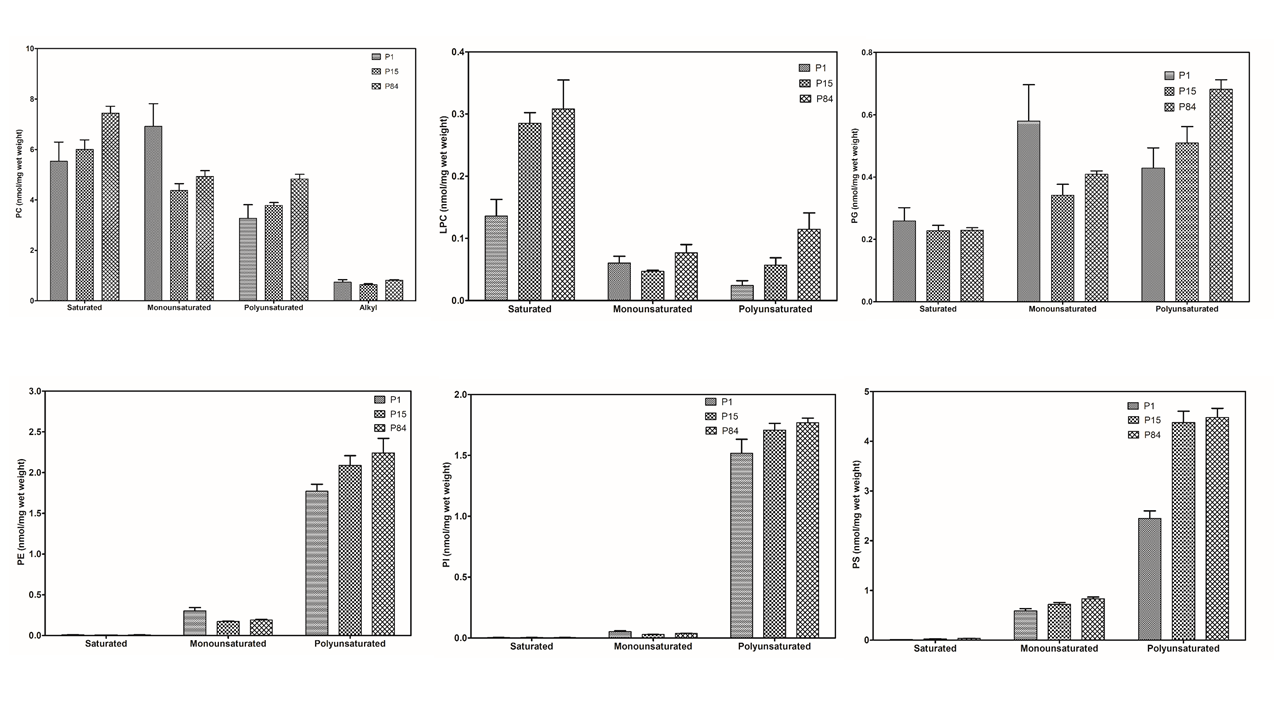

Supplement: S1 Fig — Saturated (total number of double bonds = 0), Monounsaturated (total number of double bonds = 1), Polyunsaturated (total number of double bonds ≥2) lipids. (TIF) [file pone.0203464.s004.tif]

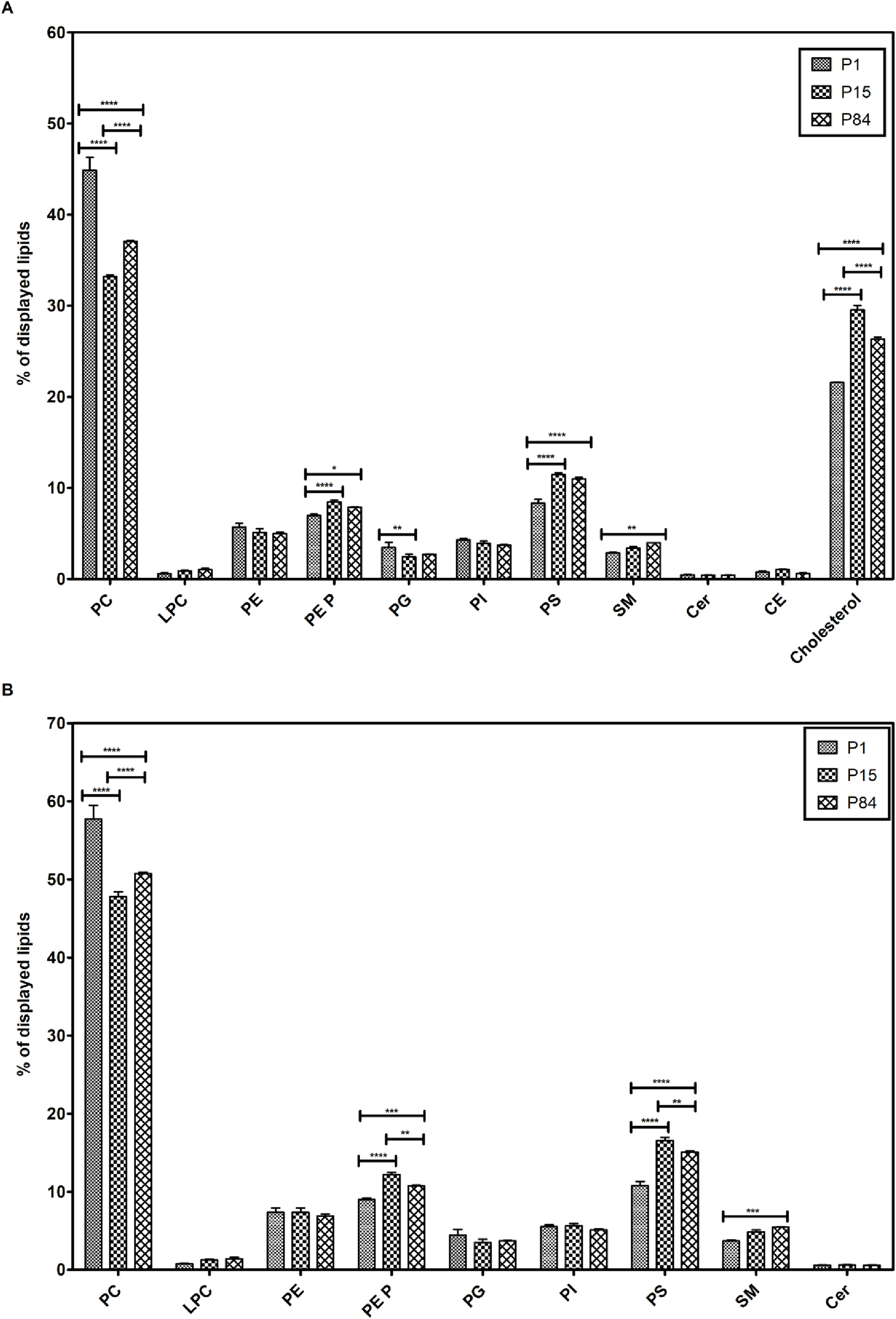

Supplement: S2 Fig — The displayed values are mol% of the respective lipid class of all analyzed lipids. Panel A) Glycerophospholipids (GP), sphingolipids (SP), cholesteryl esters (CE) and cholesterol. Panel B) only GP, SP without CE and cholesterol. Values are represented as mean ± SD, p-value summary: **** P < 0.0001, *** P < 0.001, ** P < 0.01, *P < 0.05. (TIF) [file pone.0203464.s005.tif]
